# Effect of Ensiled Mulberry Leaves and Sun-Dried Mulberry Fruit Pomace on Finishing Steer Growth Performance, Blood Biochemical Parameters, and Carcass Characteristics

**DOI:** 10.1371/journal.pone.0085406

**Published:** 2014-01-10

**Authors:** Zhenming Zhou, Bo Zhou, Liping Ren, Qingxiang Meng

**Affiliations:** 1 State Key Laboratory of Animal Nutrition, China Agricultural University, Beijing, P. R. China; 2 College of Animal Science and Technology, China Agricultural University, Beijing, P. R. China; German Institute of Human Nutrition Potsdam-Rehbrücke, Germany

## Abstract

Fifty-one Simmental crossbred steers (357.0±16.5 kg) were used to compare a standard total mix ration (TMR) with variants on animal performance, ruminal fermentation, blood biochemical parameters, and carcass characteristics. Corn grain and cotton seed meal were partially replaced by ensiled mulberry leaves (EML) or sun-dried mulberry fruit pomace (SMFP). Experimental diets had similar amounts of crude protein (CP), acid detergent fiber (ADF), and metabolizable energy (ME). Animals were divided into three groups: control group (CONT), 8% EML group, and 6.3% SMFP group. Performance, including average daily weight gain (ADG), and dry matter intake (DMI), was measured. Blood and rumen samples were collected at the end of the experiment (16 weeks). There were no differences in final body weight (*P* = 0.743), ADG (*P* = 0.425), DMI (*P* = 0.642), or ADG/DMI (*P* = 0.236) between the groups. There were no differences (*P* = 0.2024) in rumen pH values; ammonia N was lower (*P* = 0.0076) in SMFP than in the EML and CONT groups. There were differences in the concentrations of total and individual volatile fatty acids, while no differences were determined in blood biochemical parameters (i.e., plasma glucose, urea concentrations, triglycerides, total protein, insulin, IgG, alanine transaminase, and aspartate aminotransferase, *P* ≥ 0.098). No differences were observed in carcass characteristics (*P* ≥ 0.513), tenderness (*P* = 0.844), adipose and lean color values (*P* ≥ 0.149), and chemical composition (*P* ≥ 0.400); however, intramuscular fat was lower in the EML and SMFP groups compared to the CONT animals (*P* = 0.034). In conclusion, diets supplemented with these two mulberry products in an isocaloric and isonitrogenous manner have similar effects to corn grain and cotton seed meals on steer performance, blood biochemical parameters and carcass characteristics, with the exception of ruminal VFA concentrations and lower intramuscular fat content.

## Introduction

There is an increased demand for animal products in China and other developing countries, thereby increasing the demand for adequate and inexpensive animal feed. Scientists have developed feeding strategies that guarantee the sustainability of livestock production systems based on cost-effective alternatives and local feed sources. Several reports have shown that food and agricultural byproducts can be used as animal feed to replace cereal-based concentrates without negatively affecting animal production performance, such as ensiled and dried apple pomace which is used for finishing lambs [1], wet tomato pomace in lactating dairy cows [2], and tomato and cucumber waste fruits in dairy goats [3]. Mulberry trees are fast-growing deciduous plants, which are cultivated in many regions of the world. In China, mulberry cultivation areas are estimated to cover more than 10^6^ ha [4], and the biomass yield of fresh mulberry leaves is approximately 25∼30 tonnes/ha/year. Mulberry has been used as feed for silk worms for hundreds of years. Furthermore, it is used in Chinese herbal medicine for hyperlipemia and diabetes treatments due to its antioxidant, antimicrobial, and anti-hyperlipidemic properties [5]–[8]. Studies have also shown that mulberry trees are potential protein sources for herbivores [9], [10]. The addition of mulberry leaves to lamb feed reduces the need for expensive protein supplements [11]. One previous study on the rumen and gastrointestinal tract digestibility of mulberry leaves showed that it had similar digestible energy and crude protein values as alfalfa hay [12]. Compared with dry processing, mulberry leaf silage has several advantages for animal feeding. For example, the weather during harvesting need not be as fair and dry as when dried mulberry leaves are harvested. During fermentation, the silage bacteria acts on carbohydrates in the leaves to produce lactic, acetic, propionic, and butyric acid, which act as natural preservatives for mulberry leaves. This preservative method is particularly important during winters, when green forage is unavailable.

Mulberry pomace, a byproduct of the production of mulberry juice, which consists mainly of peels and stems, accounts for approximately 8% of the fresh weight of the mulberry. Typically, mulberry pomace is used as a soil enhancer or is otherwise dumped in landfills, which contributes to environmental problems. The results of our previous study revealed that mulberry fruit pomace is an excellent potential feed source for ruminal animals [13]. Even though several studies have focused on the nutritive and antioxidant value of mulberry leaves and mulberry fruit pomace, little is known about the effects of mulberry leaves and mulberry fruits on the blood biochemical parameters and carcass characteristics of finishing steers.

Our hypothesis is that EML and SMFP can be used in the diets for finishing steers without negatively affecting their productive performance. To validate this assumption, we evaluated the effects of feed supplementation with these two mulberry products in an otherwise isocaloric and isonitrogenous diet. Corn grain and cotton seed meal were partially replaced by EML or SMFP, and the performance, rumen fermentation, blood biochemical parameters, and carcass characteristics of finishing steers was assessed.

## Materials and Methods

### Animals and Diets

The animals used in this study were handled in strict accordance with the Regulations for Laboratory Animals of Beijing. The protocol was approved by the Animal Welfare Committee of the China Agricultural University (Permit Number: DK1008). In this study, 51 medium-frame crossbred Simmental steers (357.0±16.5 kg; 15 months old) were used and housed in a tie-stall facility. An adjustment period of 2 weeks allowed the steers to become acclimated to routine feeding and to allot time for proper diet adjustment before the experiment. During the adjustment period, experimental diets were gradually fed to the animals. At the end of the 2-week period, the animals were fed the experimental diets. The basal diet met the NRC (2000) requirements for 350-kg beef cattle with weight gain of 1.2 kg/d.

Mulberry leaves were harvested from a farm (Beijing, China) during July, 2010. The harvested mulberry material was ensiled without additives after chopping. The mulberry silage was then used for the feeding experiment after being stored for 50 days; mulberry fruit pomace was purchased from a local company (Guosen Co., Beijing, China). The chemical composition and silage fermentation characteristics of EML or SMFP are shown in Table S1. The animals were divided into three treatment groups: the CONT group (n = 17), which received standard TMR; the EML group (n = 17), which received a dietary ingredient of ensiled mulberry leaves; or the SMFP group (n = 17), which received a dietary ingredient of sun-dried mulberry fruit pomace (Table 1). Animals were fed twice daily at approximately 0800 and 1700 and had ad libitum access to water and a trace mineral salt block. Diet refusals were recorded every day; the amounts of feed were adjusted every 3 to 4 d to maintain the preferred daily diet refusal rate of 5∼10%. Steer body weight was monitored to assess growth performance during the 16-week trial period.

**Table 1:** Ingredients and nutrient composition of the experimental diets.

Item	Experimental diet^1^		
	CONT	EML	SMFP
**Ingredient composition, %(DM basis)**			
Corn silage	40.0	40.0	40.0
Brewer’s grain	20.0	20.0	20.0
Corn	30.2	27.0	24.5
Cotton seed meal	7.8	3.0	7.2
Ensiled mulberry leaves	0.0	8.0	0.0
Sun-dried mulberry fruit pomace	0.0	0.0	6.3
Limestone powder	0.7	0.7	0.7
Calcium hydrogen phosphate	0.1	0.1	0.1
Sodium bicarbonate	0.5	0.5	0.5
Salt	0.5	0.5	0.5
Premix^2^	0.2	0.2	0.2
**Nutrient composition, DM basis**			
ME (Mcal/kg)	2.69	2.69	2.70
CP (%)	14.01	14.06	13.98
Ca (%)	0.56	0.57	0.55
P (%)	0.33	0.33	0.33
ADF (%)	21.42	22.56	21.51

^1^ CONT  =  control; EML  =  ensiled mulberry leaves; SMFP  =  sun-dried mulberry fruit pomace.

^2^ Supplied per kilogram of dietary DM: 15 mg of Cu, 65 mg of Zn, 28 mg of Mn, 0.7 mg of I, 0.2 mg of Co, 0.3 mg of Se, 6,000 IU of vitamin A, 600 IU of vitamin D, and 47 IU of vitamin E.

^3^ ME  =  metabolizable energy; CP  =  crude protein; ADF  =  acid detergent fiber.

### Sample Collection and Laboratory Analysis

Samples of dietary ingredients (EML and SMFP), three experimental diets, and diet refusals were collected every 2 weeks, dried at 72°C in an air oven, and ground in a Wiley Mill (A. H. Thomas Co., Philadelphia, PA) through a 1-mm screen. The dried samples were stored for chemical and nutrient content analysis. Briefly, samples were analyzed for amylase-treated ADF using the Ankom200 Fiber Analyzer (Ankom Technology Corp., Macedon, NY) according to previous study [14]; heat stable amylase (Ankom Technology Corp.) and sodium sulfite (Fisher Scientific, Waltham, MA) were used. Nitrogen content of three treatments and diet refusals were analyzed with a Nitrogen Analyzer (Model Rapid N III, Elementar, Analysis Systems GmbH, Germany); 6.25 was used as the conversion factor to obtain crude protein (CP) values. When there were differences in the nutritional values of the diet between the measured and initial values, the dietary formula was adjusted according to the measured nutrient values.

Blood samples (10 mL) were collected 2 hours after morning feeding at 10:00 on the final feeding week by jugular venipuncture into a single 10-mL vacuum tube containing Na-heparin. Samples were placed on ice for transport to the laboratory and centrifuged at 5,000 × g for 20 min at 4°C, and collected plasma were frozen at –20°C until analysis. Plasma concentrations of glucose, urea, triglycerides, total protein, alanine transaminase, and aspartate aminotransferase were determined using an automated enzymatic colorimetric principle on a Cobas Integra 400 Instrument (Roche Diagnostics) as described in our previous study [15].

After the steers were slaughtered, samples (approximately 500 mL, mix of liquid and solid) from the dorsal, central, and ventral regions of the rumen were collected to form one composite sample, and then strained the sample through four layers of cheesecloth for rumen fluid collection. The pH of the rumen fluid was measured immediately using a Model PHS-3C pH meter (Shanghai Leici Scientific Instrument Co., Ltd., China). To the filtrates (10 mL), 1 mL of 50% (v/v) HCl was added for ammonia N analysis, and 1 mL of a metaphosphoric acid (187.5 g/L) and formic acid (250 mL) solution was added for volatile fatty acids or VFA determination [16]. Ammonia N concentration in the filtrates was determined with a UV-VIS 8500 spectrophotometer (Shanghai Tianmei Scientific Instrument Co., Ltd., China). VFAs were quantified using a gas liquid chromatograph (GLC, 6890 N model, Agilent Technologies) with a capillary column (30 m×0.32 mm *i.d*.; 0.50 µm phase thickness) [17].

### Steer Slaughter and Carcass Measurements

At the end of feeding trail, steers were starved for 24 h before they were loaded (at 0600 h) and transported 5 km to a commercial slaughterhouse. Efforts were made to minimize the suffering of steers. Briefly, wide ramps with slopes (11°) were used for the loading and unloading steers, and transport vehicles were equipped with non-slip flooring. Once a vehicle arrived at slaughterhouse, efficient scheduling procedures were implemented to ensure that steers were unloaded quickly. Pre-slaughter handling systems were used to encourage the smooth movement of stock. When steers were slaughtered, they were restrained in an upright position with their head held fast and the neck exposed in a suitable position for the incision of the throat. The knife used for cattle has a long, extremely sharp, and undamaged blade. The intention was to produce the immediate outpouring of blood by severing both jugular veins and both carotid arteries. Steer final BW was recorded prior to slaughter to determine dressing percentage. Hot carcass weight was obtained on the day of slaughter; carcasses were subsequently stored at 4°C for 48 h. Cold carcass weights and carcass shrink weights were measured. Carcasses were cut between the 12^th^ and 13^th^ ribs for assessment of carcass quality attributes and yield grade parameters. Carcass quality attributes included 12^th^-rib fat thickness, longissimus muscle (LM) area, intramuscular fat, and ultimate pH values. Carcass color was assessed by measuring L*, a*, and b* color values using a portable Minolta chromameter (Minolta Chroma Meter CR-400 colorimeter, Minolta Corp., Osaka, Japan) on the cut lean surface and carcass external fat along the lateral side of the carcass. Color readings were recorded in the L* (0 = black, 100  =  white), a* (negative values  =  green, positive values  =  red), and b* (negative values  =  blue, positive values  =  yellow) color space (CIELAB); large pieces of connective tissue and intramuscular fat were avoided. Color saturation was calculated as described by the operational manual [18].

### Meat Measurements

Warner-Bratzler shear force (WBSF) values were determined according to the AMSA (1995) guidelines. Following carcass data collection, strip loins were excised from the left side of each carcass, vacuum-packaged, and aged for 14 d at 4°C. After the aging period, 2.54-cm-thick steaks were made, vacuum packaged and frozen (–20°C) for later analysis. Steaks for WBSF were thawed for 24 h at 4°C and cooked to an internal temperature of 71°C in an open-hearth electric broiler (George Foreman GGR50, Salton Inc., Lake Forest, IL) with thermocouples inserted to approximately the geometric center of the steak. Cooking loss, expressed as a percentage of weight loss, was calculated from the ratio between the initial weight (before cooking) and the final weight (after cooking). At least six 1.27-cm-diameter pieces were removed from each steak parallel to the muscle fiber orientation [19]. The pieces were sheared perpendicular to the muscle fiber orientation using a shear device (Warner-Bratzler Meat Shear 2356X). Peak shear force values were expressed in kg; six pieces were analyzed.

Meat samples were trimmed of external fat and connective tissue for determination of ash, protein, moisture, and ether-extractable lipid. Briefly, crude protein content was calculated from the nitrogen content (%N × 6.25) analyzed by a Nitrogen Analyzer (Model Rapid N III, Elementar, Analysis Systems GmbH, Germany); ash content was determined by heating the steak sample at 550°C for 15 h; moisture content was determined by weight loss after freeze-drying at –55°C for 5 d; and lipid content was determined by the Ankom procedure (Am 5-04, AOCS, 2004). Analyses were performed in duplicate and mean values were used for statistical analysis.

### Statistical Analyses

Data for animal growth performance, rumen fermentation, blood biochemical parameters, and carcass characteristics were analyzed using the GLM procedure (SAS Inst. Inc., Cary, NC). Diet was considered as a fixed effect in this model, and the animal was considered as random effect. Statistical differences between the mean values were tested using Duncan’s multiple range test. Significant effects of the treatment were declared at *P*<0.05.

## Results and Discussion

### Feed Intake and Growth Performance

The chemical composition of the experimental diets is shown in Table 1. Crude protein, energy, and ADF contents were similar in all experimental diets. The low NDF and high crude protein contents in mulberry leaves make this forage a palatable feed for ruminants [20]. Therefore, silage mulberry leaves could improve livestock productivity by improving digestibility. Previous studies have shown that mulberry leaves benefit animal health and growth performance. Several bioactive compounds in mulberry leaves, including 1-deoxynojirimycin, γ-aminobutyric acid, and phenolic compounds, have strong antioxidant properties [21]. Additionally, mulberry fruits have antioxidative and anti-inflammatory properties [22], which suggests that mulberry silage and sun-dried mulberry fruits may be useful as a functional feed for improving the growth performance and health status of beef cattle.

The DMI, ADG, and FCR of the Simmental crossbred steers are summarized in Table 2. Ensilaged mulberry leaves had the same effect as the corn grain and cotton seed meal they replaced on animal growth performance (*P* > 0.1) after 16 weeks, which indicated that EML and SMFP could be used in finishing steers without negative effects on animal growth performance. Our results are similar to previous study, which reported that the partial replacement of cereals with mulberry leaves (*P*<5%) had no effects on the DMI of ruminant animals [23]. Furthermore, there were no negative effects on ADG in growing cattle when cotton seed was partially replaced with fresh mulberry leaves [9]. Taking into account the feed cereal amount, average feed intakes, and current costs, a reduction of 5.65% and 5.94% in feeding costs may be achieved by using diets that consist of EML or SMFP, respectively (unpublished data).

**Table 2:** Growth performance of finishing steers fed a total mixed ration supplemented with ensiled mulberry leaves (EML) or sun-dried mulberry fruit pomace (SMFP).

Item	Experimental diet^1^			SEM	*P*-value
	CONT	EML	SMFP		
Animal number	17	17	17		
Initial body weight (kg)	353.6	360.5	356.75	5.60	0.812
Final body weight (kg)	500.2	495	491	11.68	0.743
ADG (kg/d)	1.22	1.19	1.20	0.14	0.425
DMI (kg/d)	7.96	8.15	8.29	0.211	0.642
ADG/DMI	0.153	0.141	0.145	0.083	0.236

^1^ CONT  =  control; EML  =  ensiled mulberry leaves; SMFP  =  sun-dried mulberry fruit pomace.

### Rumen Fermentation

The variables of rumen fermentation are summarized in Table 3. There were no differences (*P*  =  0.2024) in the pH values of rumen fluid between the treatment groups. The rumen ammonia N concentration was lower (*P*  =  0.0076) in the SMFP group than in the EML and CON groups. The VFA concentration in the rumen fluid was higher (*P*  =  0.0427) in the EML and CON groups than in the SMFP group. There were differences in the concentrations of individual fatty acids between the treatment groups. The concentration of acetate was higher (*P*  =  0.0015) in the SMFP group than in the CONT or EML groups. The concentration of propionate was lower (*P*  =  0.0085) in SMFP than in CONT. The ratio of acetate to propionate was higher (*P*  =  0.0022) in SMFP than in CONT and EML; however, the difference between CONT and EML was not statistically significant. There were no significant differences between the treatment groups in the concentrations of butyrate (*P*  =  0.7361). The concentrations of isobutyrate, valerate, and isovalerate were lower *(P* ≤ 0.0466) in SMFP than in CONT and EML; however, the difference between CONT and EML was not statistically significant. When animals are fed high concentrate diets, the amount of propionate produced increases relative to the acetate concentration, which results in increased glucose production and more available net energy for the animals. Increased propionate production also increases insulin secretion, which increases fat and protein synthesis and decreases fat and protein degradation [24].

**Table 3:** Rumen fermentation of finishing steers fed a total mixed ration supplemented with ensiled mulberry leaves (EML) or sun-dried mulberry fruit pomace (SMFP).

Item	Experimental diet^1^			SEM	*P*-value
	CONT	EML	SMFP		
pH value	5.51	5.65	5.71	0.08	0.2024
Ammonia N (mg/dL)	17.82^a^	17.93^a^	11.95^b^	1.44	0.0076
Total VFA (mM)	83.16^a^	83.87^a^	70.65^b^	3.88	0.0427
Individual VFA (mM/100 mM))					
Acetate	68.26^b^	69.13^b^	70.78^a^	0.43	0.0015
Propionate	20.07^a^	19.40^a,b^	18.57^b^	0.30	0.0085
Isobutyrate	0.77^a,b^	0.80^a^	0.63^b^	0.05	0.0466
Butyrate	8.14	7.99	7.86	0.25	0.7361
Isovalerate	1.38^a^	1.43^a^	1.12^b^	0.06	0.0053
Valerate	1.38^a^	1.25^a^	1.04^b^	0.05	0.0008
Acetate/Propionate	3.41^b^	3.58^b^	3.82^a^	0.07	0.0022

^1^ CONT  =  control; EML  =  ensiled mulberry leaves; SMFP  =  sun-dried mulberry fruit pomace.

^a,b^ Values in the same row with different superscripts are significantly different (*P*<0.05).

SMFP and EML provided carbohydrates for ruminal bacteria, which utilized more ammonia N for microbial growth, and consequently reduced the amount of free ammonia N in the rumen (Table 3). No information is available on the effect of EML or SMFP on rumen fermentation. Our results revealed that SMFP decreased ruminal ammonia N concentrations and increased acetate/propionate ratios, indicating that SMFP has the ability to modify rumen fermentation and bacterial composition, and improve the efficiency of N metabolism in the rumen. Studies have reported that mulberry fruits are rich in polyphenols [25]. The presence of condensed tannins in the diet reduce soluble protein in the rumen and rumen ammonia N concentration, but increase non-ammonia N outflow from sheep rumen [26], [27]. We proposed that polyphenols in SMFP had similar effects on protein degradation and non-ammonia N outflow.

### Blood Biochemical Parameters

The effects of EML and SMFP on the blood biochemical parameters are shown in Table 4. There were no differences in plasma urea nitrogen between EML, SMFP, and CONT (*P* > 0.05). Plasma urea nitrogen is the end product of proteolysis and amino acid metabolism; its concentration is dependent on the crude protein level. Additionally, plasma urea nitrogen is negatively correlated with body nitrogen deposition and the utilization rate of proteins. High levels of casein in the diet increase plasma urea-N concentrations, whereas supplemental energy or glucose decrease plasma urea-N concentrations [28]-[30]. The lack of differences in plasma urea-N concentrations between the treatment groups indicates that there was probably no amino acid deficiency or imbalance in the EML and SMFP groups. Insulin is an anabolic hormone with key roles in glucose [31], [32], lipid [33], and protein metabolism [34]. The stabilization of plasma insulin reflects a balance in protein and amino acid metabolism. There were no significant differences in plasma insulin concentrations between the three groups (*P*  =  0.491; Table 4), indicating that EML and SMFP had the similar effect as corn grain and cotton seed meal on amino acid metabolism in steers. A previous study reported that insulin regulates feed intake and nutrient partitioning in ruminants [35]. Our result of the same level of insulin concentration is consistent, with no differences found in the DMI between the three treatments. Plasma glucose, albumin, cholesterol, triglycerides, HDL, LDL, and VLDL were not affected by the supplemented feeds (*P* > 0.05). Additionally, alanine transaminase and aspartate aminotransferase concentrations were not affected by treatments (*P* > 0.05), which indicated the absence of hepatic or cardiac dysfunction in the animals.

**Table 4:** Blood biochemical parameters of finishing steers fed a total mixed ration supplemented with ensiled mulberry leaves (EML) or sun-dried mulberry fruit pomace (SMFP).

Item	Experimental diet^1^			SEM	*P*-value
	CONT	EML	SMFP		
Blood urea N (mmol/L)	3.85	3.41	3.67	0.055	0.469
Insulin (μU/mL)	1.35	1.46	1.45	0.021	0.491
Total protein (g/L)	78.34	79.57	74.21	0.044	0.856
Albumin (g/L)	32.87	34.89	32.42	0.190	0.696
Glucose (g/L)	5.21	5.35	5.11	0.456	0.143
Cholesterol (mmol/L)	4.21	4.02	4.34	0.267	0.456
Triglyceride (mmol/L)	0.33	0.31	0.34	0.493	0.098
HDL (mmol/L) 2	2.64	2.41	2.35	0.562	0.251
LDL (mmol/L) 2	0.95	1.13	0.98	0.345	0.667
VLDL (mmol/L) 2	1.45	1.62	1.51	0.876	0.811
Alanine transaminase (U/L)	30.64	29.96	30.11	0.454	0.349
Aspartate aminotransferase(U/L)	35.11	36.23	35.64	0.659	0.289
IgG (g/L)	11.50	12.34	12.62	0.516	0.538

^1^ CONT  =  control; EML  =  ensiled mulberry leaves; SMFP  =  sun-dried mulberry fruit pomace.

^2^ HDL: High density lipoprotein; LDL: Low density lipoprotein; VLDL: Very low density lipoprotein.

The addition of agricultural byproducts, including bioactive compounds, to animal feed affects the metabolic activity of finishing steers. For example, grape pomace modulate metabolic syndrome [36], wine byproducts lower plasma triacylglycerol and phospholipid concentrations [37]. The chronic intake of bioactive compounds can affect animal performance. Silage mulberry leaves and fruits contain several bioactive compounds, such as phenolic and flavonoid compounds [22], [25], which increase humoral immunity, cell mediated immunity, and activate macrophage activity [38]. However, the results of our study showed no differences in blood metabolism characteristics between the treatment groups could be attributed to two possible reasons: i) processing conditions of fermentation and sun-drying could reduce or degrade the bioactive compounds; and ii) the supplementation of <10% of the feed could not initiate obvious downstream effects in animal physiological systems.

### Carcass Characteristics

The carcass characteristics of the three experimental groups are shown in Table 5. There were no differences between the EML, SMFP, and CONT groups (*P* ≥ 0.513 to *P* ≥ 0.964). Mean LM area ranged from 76.48 cm^2^ (CONT) to 60 cm^2^ (SMF); mean fat thickness over the 12^th^ rib ranged from 0.29 cm (SMF) to 0.31 cm (CONT). Similar carcass characteristics revealed that there were no adverse effects by EML or SMFP. There were no differences in dressing percentage between EML, SMFP, and CONT (*P*  =  0.973). Dressing percentage was 54.14% in EML and 54.89% in SMFP. Hot carcass weight and cold carcass weight were not affected by the treatments. Switching from a conventional diet to a partially substituted diet can change the amount of fat deposited in finishing steers, thereby affecting profitability and consumer acceptance. Energy density and feeding intensity are the main factors that affect carcass and intramuscular fatty acid composition [39]. Modifying diet energy density and increasing the supply of unsaturated fatty acids affect muscle fatty acid composition [40]. Although the three treatment groups had similar metabolizable energy (ME) in their diets, intramuscular fat content was lower in the EML and SMFP groups than in the CONT group (*P*  =  0.034). The lower propionate concentrations in the rumen of the SMFP group probably contributed to the lower intramuscular fat content. Different fat contents between the treatment groups could be attributed to differences in feed chemical compositions. Therefore, the EML and SMFP groups may have received plant secondary compounds with anti-lipogenesis properties.

**Table 5:** Carcass characteristics of finishing steers fed a total mixed ration supplemented with ensiled mulberry leaves (EML) or sun-dried mulberry fruit pomace (SMFP).

Item	Experimental diet^1^			SEM	*P*-value
	CONT	EML	SMFP		
BW (kg)	500.2	495	491	11.68	0.743
HCW (kg)	272.0	269.5	271.3	10.56	0.591
Cold carcass weight (kg)	271.1	268.0	270.2	9.69	0.513
Dressing percentage (%)	54.19	54.14	54.89	0.58	0.973
12^th^-rib fat thickness (cm)	0.31	0.28	0.29	1.28	0.713
LMA^2^ (cm^2^)	76.48	78.64	77.45	3.69	0.532
pH value	6.15	6.04	6.11	0.31	0.26
External fat color^3,4^					
L*	34.11	40.23	38.32	2.56	0.754
a*	15.14	18.05	27.68	1.19	0.895
b*	8.73	7.45	7.29	1.05	0.149
c*	19.52	20.07	20.10	0.93	0.256
12^th^-rib lean color^4^					
L*	81.98	80.14	80.88	2.265	0.783
a*	3.69	3.56	3.84	0.862	0.861
b*	7.73	7.98	8,07	1.614	0.715
c*	8.34	8.70	8.42	0.123	0.532

^1^ CONT  =  control; EML  =  ensiled mulberry leaves; SMFP  =  sun-dried mulberry fruit pomace.

^2^ LMA  =  LM area measured at 12^th^ rib.

^3^ Fat color measurements obtained approximately 20 cm ventrally to the lateral process of the split carcass adjacent the 13th rib.

^4^ CIE color measurements: L*  =  lightness, black (0) to white (100); positive a*  =  red; negative a*  =  green; positive b*  =  yellow; negative b*  =  blue; c* =  color saturation  =  [(a*)^2^ + (b*)^2^]^1/2^ whereby a large number is considered to be more vivid.

To our knowledge, this study is the first to evaluate the carcass characteristics of steers. Future studies should assess the effect of different levels of EML and SMFP on animal growth performance. The supplementation of finishing steer diets with either EML or SMFP did not affect WBSF values (*P*  =  0.844). Carcass color and other meat quality characteristics are shown in Table 5. There were no differences in lean L* values (*P*  =  0.783), a* values (*P*  =  0.861), b* values (*P*  =  0.715), or c* values (*P* = 0.532) between treatment groups. Similarly, there were no differences in adipose L* values (*P* = 0.754), a* values (*P* = 0.895), b* values (*P* = 0.149), or c* values (*P* = 0.256) between treatment groups. There were no differences in pH values of the 48-h LM between the treatment groups; pH values were within the normal pH range (Table 5). There were no significant differences in the color values of adipose and lean tissues; therefore, EML and SMFP did not affect adipose and lean tissue color in steers.

Table 6 shows the effect of the different treatment groups on moisture, protein, intramuscular fat, and ash percentages of steaks. Moisture, protein, and ash percentages were similar between the three treatment groups; however, intramuscular fat content was significantly higher in the CONT group than in the EML and SMFP groups. A previous study showed that WBSF values are negatively correlated with marbling scores and intramuscular fat content, indicating that as intramuscular fat decreases, WBSF values increase and steaks become tougher [19]. However, our results revealed that there were no differences in WBSF values, even at the lowest intramuscular fat content, in the EML and SMFP groups. Intramuscular fat plays an integral role in steak tenderness; high intramuscular fat contents are associated with increased beef tenderness. Even though there were no differences in tenderness or flavor, low intramuscular fat contents affect juiciness, flavor, and sensory attributes.

**Table 6:** WBSF and chemical composition of steak samples of finishing steer fed a total mixed ration supplemented with ensiled mulberry leaves (EML) or sun-dried mulberry fruit pomace (SMFP).

Item	Experimental diet^1^			SEM	*P*-value
	CONT	EML	SMFP		
WBSF^2^ (kg)	4.78	4.75	4.34	0.61	0.844
Cooked rate (%)	65.72	66.89	68.13	2.53	0.961
Drip loss (%)	6.89	7.12	6.93	1.24	0.922
Moisture (%)	75.49	75.82	75.65	0.37	0.146
Protein (%, DM basis)	87.24	87.62	86.82	0.42	0.276
Intramuscular fat (%, DM basis)	5.62^a^	4.53^b^	4.62^b^	0.34	0.034
Ash (%, DM basis)	5.36	5.74	5.78	0.21	0.467

^1^ CONT  =  control; EML  =  ensiled mulberry leaves; SMFP  =  sun-dried mulberry fruit pomace.

^2^ WBSF  =  Warner-Bratzler shear force values of tenderness.

^a,b^ Values in the same row with different superscripts are significantly different (*P*<0.05).

## Conclusion

This study compared steer performance variables between those fed with a standard TMR and those fed with diets in which corn grain and cotton seed meal were partially replaced by EML or SMFP. Diets that included these two mulberry products had similar effects as the corn grain and cotton seed meal on general performance, blood biochemical parameters, and carcass characteristics. The supplemented feeds did produce variable levels of ruminal VFAs and lower intramuscular fat content.

## Supporting Information

Table S1 Chemical composition and silage fermentation characteristics of ensiled mulberry leaves (EML) or sun-dried mulberry fruit pomace (SMFP).
(DOC)
