# Supplementary material for: Effect of Ensiled Mulberry Leaves and Sun-Dried Mulberry Fruit Pomace on Finishing Steer Growth Performance, Blood Biochemical Parameters, and Carcass Characteristics
Source: PLoS One. 2014 Jan 10;9(1):e85406. doi: 10.1371/journal.pone.0085406 (PMC3888424; doi:10.1371/journal.pone.0085406)
Supplement: Table S1 — Chemical composition and silage fermentation characteristics of ensiled mulberry leaves (EML) or sun-dried mulberry fruit pomace (SMFP). (DOC) [file pone.0085406.s001.doc]

Table S1. Chemical composition and silage fermentation characteristics of ensiled mulberry leaves (EML) or sun-dried mulberry fruit pomace (SMFP)

| Item | EML | SMFP |
| --- | --- | --- |
| Chemical composition |  |  |
| DM (g/kg) | 365.2 | 860.4 |
| OM (g/kg DM) | 873.5 | 870.5 |
| CP (g/kg DM) | 189.6 | 220.3 |
| NDF (g/kg DM) | 401.3 | 368.8 |
| ADF (g/kg DM) | 250.2 | 491.3 |
| Fermentation characteristics |  |  |
| pH value | 4.2 | / |
| Lactate (g/kg DM) | 242.2 | / |
| NH3-N (g/kg total N) | 126.3 | / |
| Acetate (g/kg DM ) | 10.5 | / |
| Propionate (g/kg DM ) | 2.5 | / |
| Butyrate (g/kg DM ) | 1.0 | / |

EML = ensiled mulberry leaves; SMFP = sun-dried mulberry fruit pomace.
